# Supplementary material for: Invasive Fungal Infection Caused by Magnusiomyces capitatus in an Immunocompromised Pediatric Patient with Acute Lymphoblastic Leukemia in Mexico City: A Case Report
Source: J Fungi (Basel). 2022 Aug 15;8(8):851. doi: 10.3390/jof8080851 (PMC9410127; doi:10.3390/jof8080851)
Supplement: Supplementary file 1 [file jof-08-00851-s001.zip › jof-1835709-supplementary.pdf]

Table S1. Pairwise levels of identity of *M. capitatus* ENCB-HI-834 with their closely related species, by using of ITS and 28S markers.

**ITS**	**1**	**2**	**3**	**4**	**5**	**6**	**7**	**8**	**9**	**10**	**11**	**12**
1. ***Magnusiomyces capitatus* ENCB-HI-834 (MN832904)**												
2. *Magnusiomyces capitatus* H2-5 (KT876611)	100											
3. *Magnusiomyces capitatus* SGX1001 (MH999810)	100	100										
4. *Magnusiomyces capitatus* Dmin 164990 (MG241530)	100	100	100									
5. *Magnusiomyces capitatus* H4-3 (KT876612)	100	100	100	100								
6. *Yarrowia lipolytica* CBS 6124 (NR111212)	56	56	56	56	56							
7. *Candida phangngaensis* ATCC MYA4467 (NR111357)	57.1	57.1	57.1	57.1	57.1	78						
8. *Groenewaldozyma salmanticensis* MUCL 29881 (NR111299)	65	65	65	65	65	57	64					
9. *Debaryomyces hansenii* JCM 1990 (NR120016)	59.2	59.2	59.2	59.2	59.2	55.5	60.4	66.4				
10. *Clavispora lusitaniae* CBS 6936 (NR130677)	59.6	59.6	59.6	59.6	59.6	65.3	71.1	64.7	62.3			
11. *Lodderomyces elongisporus* ATCC 11503 (NR111593)	60.5	60.5	60.5	60.5	60.5	59.2	62.5	68.4	76.6	67.1		
12. *Candida orthopsilosis* ATCC 96139 (NR130661)	63	63	63	63	63	61.6	64.4	69.9	79.1	68.5	88.5	

**28S**	1	2	3	4	5	6	7	8	9	10	11	12
1. ***Magnusiomyces capitatus* ENCB-HI-834 (MN833644)**												
2. *Magnusiomyces capitatus* CBS 162.80 (NG055400)	100											
3. *Magnusiomyces capitatus* CBS 580.82 (KU301175)	100	100										
4. *Magnusiomyces capitatus* CBS 312.76 (KU301159)	100	100	100									
5. *Magnusiomyces capitatus* CBS 562.97 (KU301164)	100	100	100	100								
6. *Magnusiomyces capitatus* CBS 579.82 (KU301174)	100	100	100	100	100							
7. *Magnusiomyces capitatus* CBS 12994 (KU301154)	99.6	99.6	99.6	99.6	99.6	99.6						
8. *Groenewaldozyma salmanticensis* NRRL Y-17090 (NG055414)	70.1	70.1	70.1	70.1	70.1	70.1	69.7					
9. *Yarrowia lipolytica* NRRL YB-423 (NG055393)	68.6	68.6	68.6	68.6	68.6	68.6	68.2	68.6				
10. *Debaryomyces hansenii* NRRL Y-7426 (NG042634)	68.4	68.4	68.4	68.4	68.4	68.4	68	77.5	70.3			
11. *Clavispora lusitaniae* NRRL Y-11827 (NG055408)	67.5	67.5	67.5	67.5	67.5	67.5	67.1	72	68.2	71.7		
12. *Lodderomyces elongisporus* ATCC 11503 (NG054825)	68.3	68.3	68.3	68.3	68.3	68.3	67.9	75.5	69.5	90.2	72	
13. *Candida orthopsilosis* ATCC 96139 (NG054816)	67.5	67.5	67.5	67.5	67.5	67.5	67.2	74.3	68.8	90.5	72.7	96.8
